# Bacterial exopolysaccharides as a modern biotechnological tool for modification of fungal laccase properties and metal ion binding

**DOI:** 10.1007/s00449-018-1928-x

**Published:** 2018-03-26

**Authors:** Monika Osińska-Jaroszuk, Magdalena Jaszek, Magdalena Starosielec, Justyna Sulej, Anna Matuszewska, Monika Janczarek, Renata Bancerz, Jerzy Wydrych, Adrian Wiater, Anna Jarosz-Wilkołazka

**Affiliations:** 1 Department of Biochemistry, Maria Curie-Sklodowska University, Akademicka 19, 20-033 Lublin, Poland; 2 Department of Genetics and Microbiology, Maria Curie-Sklodowska University, Akademicka 19, 20-033 Lublin, Poland; 3 Department of Comparative Anatomy and Anthropology, Maria Curie-Sklodowska University, Akademicka 19, 20-033 Lublin, Poland; 4 Department of Industrial Microbiology, Maria Curie-Sklodowska University, Akademicka 19, 20-033 Lublin, Poland

**Keywords:** *Rhizobiaceae*, Exopolysaccharides, Stabilization, Laccase modification, Metal ion adsorption

## Abstract

Four bacterial EPSs extracted from *Rhizobium leguminosarum* bv. *trifolii* Rt24.2, *Sinorhizobium meliloti* Rm1021, *Bradyrhizobium japonicum* USDA110, and *Bradyrhizobium elkanii* USDA76 were determined towards their metal ion adsorption properties and possible modification of *Cerrena unicolor* laccase properties. The highest magnesium and iron ion-sorption capacity (~ 42 and ~ 14.5%, respectively) was observed for EPS isolated from *B. japonicum* USDA110. An evident influence of EPSs on the stability of laccase compared to the control values (without EPSs) was shown after 30-day incubation at 25 °C. The residual activity of laccases was obtained in the presence of *Rh76*EPS and *Rh1021*EPS, i.e., 49.5 and 41.5% of the initial catalytic activity, respectively. This result was confirmed by native PAGE electrophoresis. The EPS effect on laccase stability at different pH (from 3.8 to 7.0) was also estimated. The most significant changes at the optimum pH value (pH 5.8) was observed in samples of laccase stabilized by *Rh76*EPS and *Rh1021*EPS. Cyclic voltamperometry was used for analysis of electrochemical parameters of laccase stabilized by bacterial EPS and immobilized on single-walled carbon nanotubes (SWCNTs) with aryl residues. Laccases with *Rh76*EPS and *Rh1021*EPS had an evident shift of the value of the redox potential compared to the control without EPS addition. In conclusion, the results obtained in this work present a new potential use of bacterial EPSs as a metal-binding component and a modulator of laccase properties especially stability of enzyme activity, which can be a very effective tool in biotechnology and industrial applications.

## Introduction

*Rhizobiaceae* are a very diverse family of symbiotic Gram-negative bacteria occurring in the soil as free-living microorganisms or existing in special structures inside the roots of leguminous plants, where they reduce molecular nitrogen to ammonia. Symbiotic bacteria produce exopolysaccharides (EPS), which secreted outside the cell can act as an important signal molecule in initiation of the symbiosis with leguminous plants. Furthermore, exopolysaccharides protect bacteria in their natural environment against stressful conditions such as salinization, drying, and the presence of detergents or heavy metal ions [1]. The *Rhizobium* polysaccharides described in the recent literature are species-specific branched heteropolymers consisting of a number of repeated subunits containing various monosaccharides and a non-sugar group giving these molecules their acidic nature [2]. The heterogeneity of the chemical structure of exopolysaccharides produced by different strains of *Rhizobia* is very high and involves the monosaccharide composition and size of the repeating subunit, the type of glycosidic bonds, non-sugar residues, and the degree of polymerization [3, 4].

Despite the high diversity of bacterial EPSs and their physicochemical properties, only a few of them have been really used till now in industry, e.g., xanthan from *Xanthomonas campestris*, gellan gum from *Pseudomonas elodea*, and alginate from *Pseudomonas aeruginosa* used as a thickener and stabilizer in food industry [5]. Bacterial EPSs are also used as drug carriers, bioflocculants, biosorbents, and heavy metal-eliminating factors [6]. Their capacity to bind heavy metals has been successfully applied in the bioremediation process, particularly in the metal and mining industry [7]. Recently, it has been shown that certain bacterial EPSs have also antioxidant, antitumor, and immunomodulatory properties; therefore, they can be used as pharmaceuticals [8, 9]. Suitable metal ion content in the reaction medium may also significantly modulate the activity of biocatalysts. It can eliminate the diminishing effect of some metal ions and increase their activating effect. This is one of the reasons why the presence of exopolysaccharides can modulate the native properties of enzymes and yield high stability of proteins under extreme stress conditions. EPSs of this type were used, e.g., to preserve lipase from deactivation by chemicals produced during the enzyme-catalyzed reaction [10].

Laccase (EC 1.10.3.2) is an oxidoreductase, which catalyzes the reaction of oxidation of organic substrates such as phenols, polyphenols, aniline, and some inorganic compounds through an electron transfer mechanism [11]. One of the most efficient sources of extracellular laccase is the white rot fungus *Cerrena unicolor*. The enzyme isolated from this organism was purified and their kinetic properties were partially characterized [12–14]. The addition of Cu^2+^ ions was detected to stimulate effectively the production of *C. unicolor* laccase during cultivation in biofermentors [15].

With its broad spectrum of catalyzed reactions and low substrate preference, *C. unicolor* laccase can be used as a biocatalyst in many biotechnological applications, including paper and textile industries, environmental protection, medicine, as well as in the design of biosensors [16–20]. In the construction of enzyme biosensors, fungal laccases are used most frequently. Unlike many other enzymes, laccase for catalysis does not require the presence of hydrogen peroxide or another cofactor; therefore, the construction of the biosensor is greatly simplified [21]. The stability of the enzyme is an important issue requiring consideration during the design of biosensors. Although the molecular properties and methods of purification of laccase are well known, factors influencing the stability and activity of the enzyme are still not sufficiently explored [22]. To improve the biotechnological properties of laccase, modifications of the enzymatic reaction conditions are also used.

Kucharzyk et al. studied the effects of chemical modifications of laccase, using hydrophobization, hydrophilization, and polymerization, on the thermostability of the enzyme and its stability in a wide pH range [23]. One of such modifications may be the addition of some chemicals to the reaction, which increase enzyme stability and activity. The most commonly used substances are chitosan, albumin, and mucin [24–27]. However, there have been no reports of the use of the bacterial EPSs described as modulators of laccase activity and ion binding compounds.

Based on the information described above, the first aim of this work was to investigate the modulation effect of bacterial EPSs extracted from *Rhizobium leguminosarum* bv. *trifolii* Rt24.2 (*Rh24*EPS), *Sinorhizobium meliloti* Rm1021 (*Rh1021*EPS), *Bradyrhizobium japonicum* USDA110 (*Rh110*EPS), and *Bradyrhizobium elkanii* USDA76 (*Rh76*EPS) on *C. unicolor* laccase properties. The analysis was focused on investigation of changes in optimum pH, enzyme activity, as well as kinetic and electrochemical parameters. The binding capacity of the analyzed exopolysaccharides towards magnesium and iron ions was also estimated.

## Materials and methods

### Culture strains and conditions

The bacterial strains used were *R. leguminosarum* bv. *trifolii* Rt24.2 [28], *S. meliloti* Rm1021 [29], *B. japonicum* USDA110 [30], and *B. elkanii* USDA76 [31]. The strains were grown on 79CA medium, pH 7.2 [32], containing 1% glycerol and 0.1% succinate as carbon sources. Bacterial cultures were carried out in 1-L flasks with 79CA medium incubated at 28 °C and shaken at 120 rpm until the optical density of the cultures at 600 nm was 0.9 (4 days). Next, the bacteria were separated from the supernatants by double centrifugation at 7500×*g* for 30 min., and the supernatants were used to obtain an EPS preparation.

The fungal strain *C. unicolor* C-139 (Bull. ex Fr.) Murr. was obtained from the Regensburg University culture collection and deposited in the fungal collection (FLC) of the Department of Biochemistry Maria Curie-Skłodowska University, Lublin, Poland (ITS sequence deposited in GenBank under accession number DQ056858). The fermentor-scale cultivation was conducted at 28 °C in a 2.5-L Bioflo III (New Brunswick Scientific, New Brunswick, NJ) bioreactor containing 2 L of a sterilized Lindenberg and Holm medium optimized by Janusz et al. [15]. The fermentor was inoculated with 10-day-old homogenized fungal mycelium (10% of total volume), aerated at 1 L air per minute, and stirred at 100 rpm. To prevent the formation of foam, antifoam B emulsion (Sigma, St. Louis, MO) was occasionally added.

### Laccase isolation and purification

Laccase (LAC) isolation and detection was conducted as described previously [19]. The supernatant of the culture fluid was concentrated using the ultrafiltration system Pellicon 2 Mini holder (Millipore, Bedford, MA) with an Ultracel mini cartridge (10 kD cut-off). The concentrated proteins were separated by anion exchange chromatography on a DEAE Sepharose column (2.5 × 15 cm) (pH 6.5) using a chromatographic EconoSystem (Bio-Rad, Richmond, VA) equilibrated with 20 mM Tris–HCl buffer. Proteins were eluted with a linear salt gradient (0–0.5 M NaCl) at a flow rate of 1 mL/min and detected at 280 nm. Next, protein fractions exhibiting LAC activity were collected and desalted on the Sephadex G-50 column (5.0 × 20 cm). Semi-purified laccase was lyophilized on a FreeZone 18 system (Labconco, Kansas, USA) and used for further study.

### Extraction of exopolysaccharides (EPS)

The culture supernatants were concentrated to 500 ml using a reverse osmosis column and mixed with four volumes of 96% ethanol. The fluid was shaken for 10 min and then incubated for 24 h at 8 °C for EPS precipitation. Precipitated EPSs were separated from the supernatant by centrifugation (7500×*g*, 30 min, 8 °C), dried at 20 °C to remove residual ethanol, and dissolved in 10 mL of distilled water. EPS solutions obtained from four different strains of rhizobia were stored at 4 °C and used for further studies. Two independent biological repetitions were prepared and analyzed for each strain. For each formulation, the concentration of EPS was determined with the Loewus method [33].

### Determination of sugars, proteins, and phenolic compounds

The content of total sugars in the studied EPSs was examined using the phenol–sulfuric acid method described by Dubois et al. [34]. Reducing sugars were determined with the Somogyi–Nelson colorimetric method based on the procedure described by Hope and Burns with some modifications [35]. The amounts of total carbohydrates and reducing sugars were calculated with d-glucose as a standard. The protein content was analyzed according to the Bradford method [36] using Bio-Rad reagent with bovine serum albumin as a standard. The contents of reducing sugars, total sugars, and proteins were expressed in mg/g of dry weight of the polysaccharide. The concentration of the phenolic compounds was assessed with a diazosulfanilamide substrate in the DASA test [37] with the standard curve for vanillic acid.

### Determination of laccase activity and kinetics parameters

Laccase activity was determined using syringaldazine (4-hydroxy, 3,5-dimethoxybenzaldehyde) (Aldrich, USA) as a reaction substrate [38]. The LAC activity was measured in the presence of 0.5 mM of syringaldazine in 0.1 mM citrate–phosphate buffer at pH 5.3. An absorbance increase was measured for 60 s at a wavelength *λ* = 525 nm at 20 °C. One unit of laccase activity was defined as the amount of the enzyme required to oxidize 1 µmol of the substrate per minute. The activity of LAC was expressed as U per mg of protein (U/mg).

Kinetics parameters (*K*_m_, *V*_max_, and *k*_cat_) of laccase in the presence of exopolysaccharides in comparison to the control were determined by direct regression of the Michaelis–Menten hyperbola obtained experimentally. The assays were carried out using a laccase sample and syringaldazine at a concentration that ranged from 0.25 to 0.75 mM. The kinetics parameter values were received by nonlinear curve fitting according to the Michaelis–Menten equation: *V* = (*V*_max_ *C*)/(*K*_m_ + *C*), where *V* is the laccase activity and *C* is the syringaldazine concentration. The data analysis was performed with the OriginPro 8 software (OriginLab Corporation, Northampton, MA, USA).

### FT-IR spectroscopy analysis

To determine the composition of the EPSs, complete acid hydrolysis was carried out with 4.95 N trifluoroacetic acid (TFA) at 80 °C in a heating block for 4 h. Next, the mixture was cooled, evaporated, and then analyzed using infrared spectroscopy. FT-IR spectroscopy was performed with a spectrometer (Thermo Scientific Nicolet 8700A with an FT Raman Nicolet NXR module) in the wavelength range 4000–400 cm^−1^.

### Visualization of exopolysaccharide using confocal laser scanning equipment

The lyophilized samples of the bacterial EPS were treated with a Fluorescence Brightener 28 solution to detect β-linked polysaccharides. The tested EPS (1 mg) was washed once with MQ water, centrifuged at 10,000×*g* for 5 min, and stained for 30 min with 200 µL of 25 mg Fluorescence Brightener/L. After removal of the dye solution, the precipitate was washed twice with MQ water and placed on a glass slide. Observations of the EPS samples were performed using an inverted microscope Axiovert 200M equipped with an LSM 5 Pascal head (with magnification 200×).

### Assessment of the ability of EPS to bind magnesium and iron ions

Binding analyses were performed using standardized human serum containing appropriate test substances (magnesium and iron ions). Commercial standard human serum (Alpha Diagnostics, Poland) was mixed with exopolysaccharides in a proportion of 1.5:0.5 (v/v). The final concentrations of the tested substances in the human serum were 2.86 mg/dL magnesium ions and 0.174 mg/dL iron ions. The samples were incubated for 2 and 24 h at room temperature. In the next step, after centrifugation at 10,000×*g* for 10 min, the supernatant was collected and assayed towards the relevant biochemical parameters. A sample of human serum containing distilled water instead of an exopolysaccharide probe was a comparative control. Quantitative determination of the tested substances in plasma (magnesium and iron ions) was performed using commercially available biochemical test kits (Alpha Diagnostics, Poland).

### Electrochemical experiments

Electrochemical analysis of the samples was performed using the Eco Chemie Autolab potentiostat with GPES software (GPES, version 4.9, Eco Chemie, the Netherlands). A three-electrode system with Ag/AgCl (1 M KCl) as a reference electrode, platinum wire as a counter electrode, and a glassy carbon electrode (GCE, BAS) as a working electrode were used. Naphthylated carbon nanotubes were immobilized on the surface of the working electrode using the method of physical adsorption. Synthesis of nanotubes modified with naphthyl groups was performed according to the procedure described by Stolarczyk et al. [39]. Then, using the same method, the laccase was immobilized with the polysaccharides. Before each experiment, the GCE electrode was polished with aluminum oxide powder (grain size down to 0.05 m) on a wet pad, rinsed with water and ethanol, and dried at room temperature. Measurements were carried out by immersing the working electrode in a solution of 0.2 mM ABTS. In addition, measurements with Nafion instead of polysaccharide solutions were made. The studies were carried out at the potential of 200–800 mV at temperature 22 ± 2 °C at a rate of potential change of 1 mV/sw.

### Electrophoretic detection of laccase activities

The relative laccase activity was determined using the native PAGE analysis. Electrophoretic detection was performed using 10% polyacrylamide gel (with 3% stacking gel) prepared according to the Laemmli procedure [40]. 20 µg of proteins were loaded per lane. The analysis was conducted in electrode buffer (0.025 M Tris–glycine, pH 8.3) at a constant voltage of 145 V and a temperature of 4 °C. After electrophoretic separation, the gels were washed with 0.1 M citrate–phosphate buffer (pH 5.2). The detection of laccase activity bands was carried out in the same buffer with the addition of 250 µl of a 1% guaiacol solution (Sigma Chemical Co., St. Luis, USA) at 25 °C. For visualization of protein bands, the gels were stained with a 1% Coomassie Brilliant Blue (R250) solution. After the staining procedure, gel scans were performed using a G:Box apparatus (Syngene, USA).

### Effect of bacterial exopolysaccharides on laccase activity and stability

The effect of exopolysaccharides on laccase activity was determined by measuring enzyme activity in the presence of exopolysaccharides isolated from the following strains: *R. leguminosarum* bv. *trifolii* Rt24.2 *(Rh24*EPS), *B. elkanii* USDA76 (*Rh76*EPS), *B. japonicum* USDA110 (*Rh110* EPS), and *S. meliloti* Rm1021 (*Rh1021*EPS). Laccase samples containing distilled water instead of exopolysaccharides (EPS) were the controls. The samples were prepared in microtubes by mixing the laccase solutions with equal volumes of polysaccharides in a ratio of 1: 1 (at the final concentration of 0.05%). The samples were then incubated for 15 and 30 days at 4 and 25 °C. Laccase activity was measured using the standard procedure described above with syringaldazine as a reaction substrate. Simultaneously, the effect of pH on the activity of laccase in the presence of EPS was analyzed in a pH range of 3.8–7.0 using 0.1 M citrate–phosphate buffer. The activity was determined under standard conditions.

### Statistical analysis

The data were presented as mean ± SD from three independent experiments (*n* = 3). The mean values and standard deviation were calculated using one-way ANOVA (Statgraphics Online) and next the means were compared using Tukey’s multiple range test. All data were analyzed using the Excel program (Microsoft Office 2010 package). Only values of *p* ≤ 0.05 were considered as statistically significant.

## Results

### Structures and properties of rhizobial EPS

The rhizobial strains used in our studies exhibited a variable yield of EPS production (Table 1). The *R. leguminosarum* bv. *trifolii* Rt24.2 strain was characterized by the highest yield of EPS production (1900 mg *Rh24*EPS per liter of the culture). The other tested strains, i.e., *S. meliloti* Rm1021, *B. japonicum* USDA110, and *B. elkanii* USDA76, showed a much lower production yield ranging from 185 to 270 mg EPS/L culture after 4 days.

**Table 1:** Chemical composition of bacterial exopolysaccharides: yield of EPS production; value of pH; proteins; contents of total sugars and reducing sugars, and concentration of phenolic compounds

Strain	Yield of EPS production (mg/L)^a^	pH	Proteins (mg/g)^b^	Total sugars (mg/g)^b^	Reducing sugars (mg/g)^b^	Phenolic compounds (mmol/g)^b^
*R. leguminosarum* bv. *trifolii* Rt24.2	1900	6.6	16.23 ± 1.0c	733.3 ± 2.7c	30.0 ± 1.8c	106.9 ± 1.7c
*B. elkanii* USDA76	270	8.0	13.78 ± 0.5b	272.2 ± 2.5b	9.35 ± 0.4b	38.01 ± 0.7b
*B. japonicum* USDA110	225	7.3	6.65 ± 0.1a	421.1 ± 3.1a	19.8 ± 1.8a	23.57 ± 1.5a
*S. meliloti* Rm1021	185	7.5	26.05 ± 1.1d	1170.0 ± 5.5d	53.0 ± 2.3d	186.7 ± 2.1d

All results are expressed as mean ± SD from three experiments (*n* = 3). The values within the columns followed by different letters are significantly different (*p* ≤ 0.05)

^a^Yield, of EPS production in specified culture conditions expressed as mg of EPS per 1 L of culture

^b^– per gram of dry weight of crude polysaccharide

**Fig. 1:**
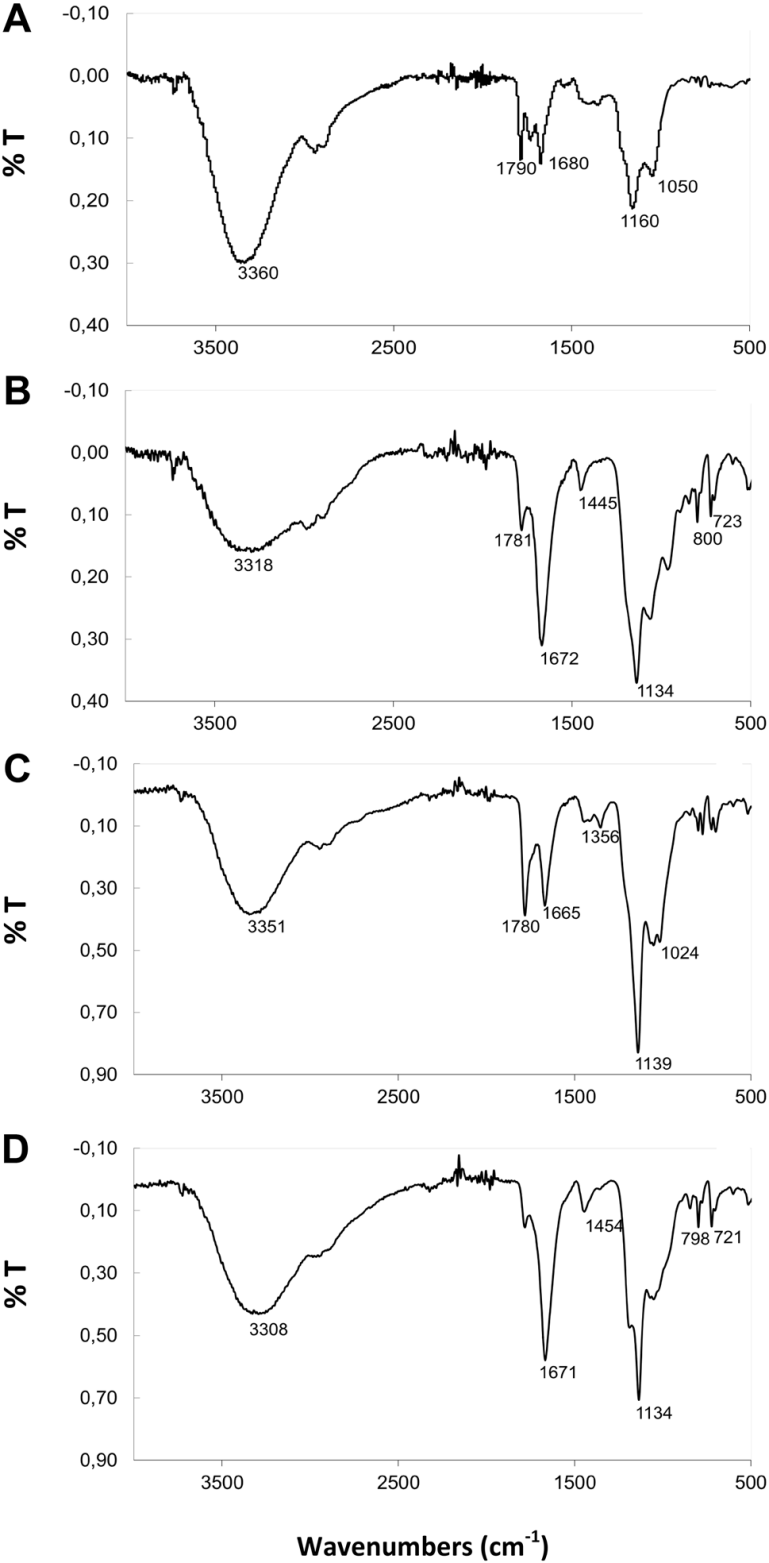
FT-IR spectra of the crude extract of hydrolyzed exopolysaccharides **a** *Rh24*EPS from *R. leguminosarum* bv. *trifolii* Rt24.2, **b** *Rh76*EPS from *B. elkanii* USDA76, **c** *Rh110*EPS from *B. japonicum* USDA110, **d** *Rh1021*EPS from *S. meliloti* Rm1021

**Fig. 2:**
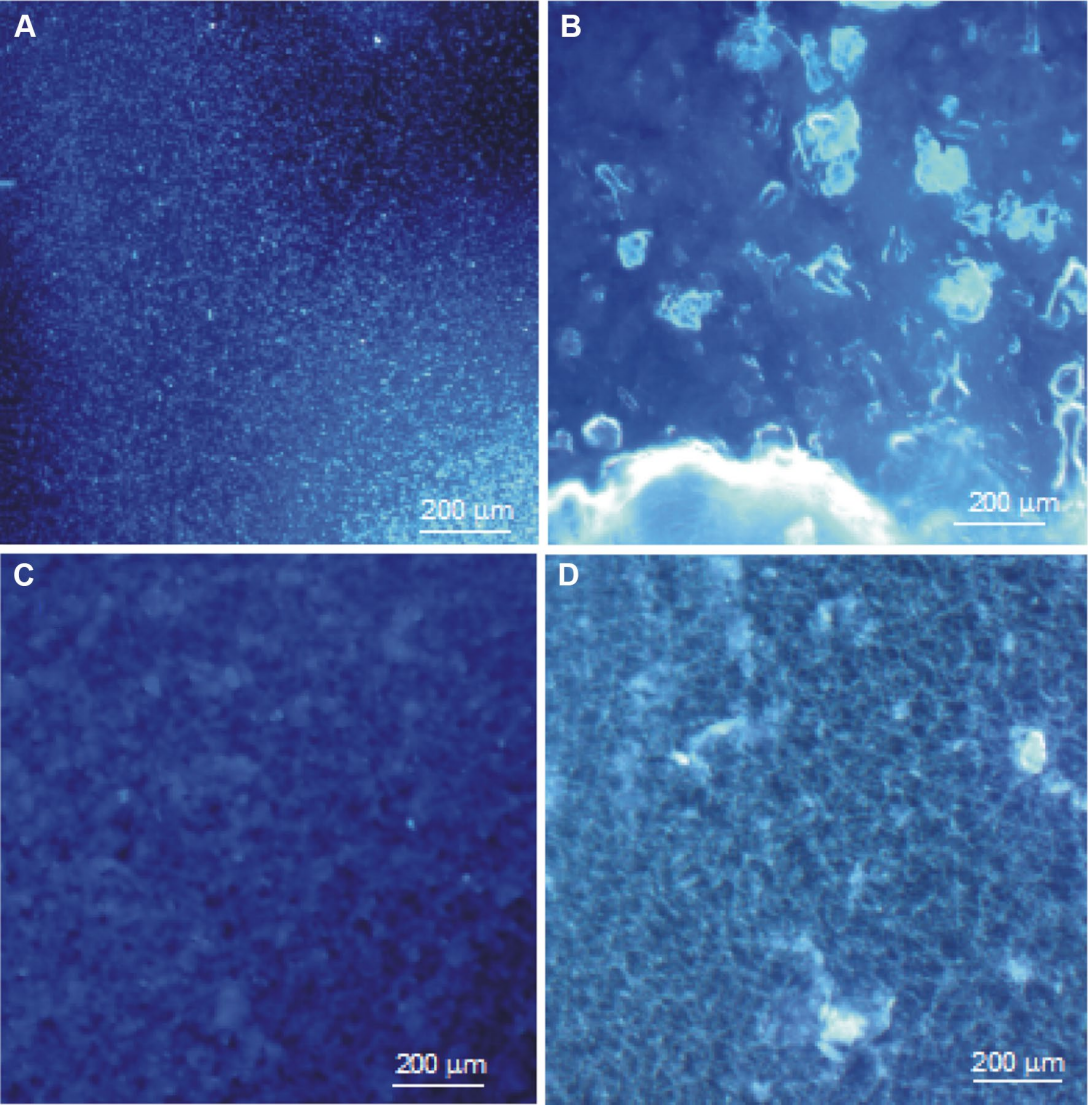
Visualization of exopolysaccharide structures using confocal laser scanning microscopy: **a** *Rh24*EPS from *R. leguminosarum* bv. *trifolii* Rt24.2, **b** *Rh76*EPS from *B. elkanii* USDA76, **c** *Rh110*EPS from *B. japonicum* USDA110, **d** *Rh1021*EPS from *S. meliloti* Rm1021

The rhizobial EPSs exhibited different chemical structures of repeating subunits. The FTIR spectroscopy technique was used for identification of functional groups in the studied polysaccharides. The FTIR spectrum of *Rh24*EPS obtained from the Rt24.2 strain showed the presence of a standard carbohydrate pattern (Fig. 1a). A signal of 3360 cm^−1^ was generated from the stretching vibration of the free –OH groups [41]. Absorption bands at 1790 and 1680 cm^−1^ are characteristic for the presence of ester bonds and proteins [42]. Additionally, the EPS spectrum showed peaks at 1160 cm^−1^ suggesting the presence of –C–O–C stretching and a band 1050 cm^−1^ indicating the presence of β-linkages in the glucosidic chain [43, 44]. Figure 1b shows the FTIR spectra of *Rh76*EPS isolated from the *B. elkanii* USDA76 strain. A band characteristic for hydroxyl groups at 3318 cm^−1^ was also present. The peaks at ca. 1672 and 1445 cm^−1^ indicate the presence of -COO^−^ deprotonated carboxylic groups [42]. The sharp band at 1134 cm^−1^ in the FT-IR spectra suggests the presence of C–O bonds [44]. Additionally, the spectrum of the extracted substances displayed peaks at 800 cm^−1^ and ca. 723 cm^−1^ indicating α-linked glycosyl residues of the main chain [45]. Similar FTIR spectra were obtained for *Rh1021*EPS isolated form the *S. meliloti* Rm1021 strain (Fig. 1d), where two bands at 798 and 721 cm^−1^ characteristic for α-linkages in the glucosidic chain were visible [45]. The FTIR spectra of *Rh110*EPS from *B. japonicum* USDA110 showed peaks at 3351 cm^−1^ characteristic for the presence of OH stretching in hydrogen bonds (Fig. 1c) [41]. The absorption bands at 1780 and 1665 cm^−1^ are attributed to the deprotonated carboxylic group (COO^−^) [42]. Furthermore, the bands at 1139 and 1024 cm^−1^ also indicate the presence of β-glycosidic bonds [43, 44]. Staining of all the tested EPS preparations using Fluorescence Brightener 28 confirmed the presence of β-linked bonds in the samples (Fig. 2). In addition, the microscopic analysis showed visible differences between the species and the heterogeneous structure of EPS produced by the investigated strains belonging to different rhizobial genera.

The chemical compositions of EPSs obtained from the tested strains are presented in Table 1. The available data showed that *Rh1021*EPS isolated from the *S. meliloti* Rm1021 strain contained the highest level of total sugar (1170 mg/g of dry weight of crude EPS) among all the tested strains. An almost fourfold lower content of total sugars was detected in *Rh76*EPS from *B. elkanii* USDA76. The concentrations of phenolic compounds and proteins in *Rh1021*EPS extracted from *S. meliloti* Rm1021 were also the highest among all the tested strains and amounted to, respectively, 186.7 ± 2.1 and 26.05 ± 1.1 mmol per 1 g of dry weight of crude polysaccharides.

### Assessment of the ability of EPS to bind magnesium and iron ions

**Fig. 3:**
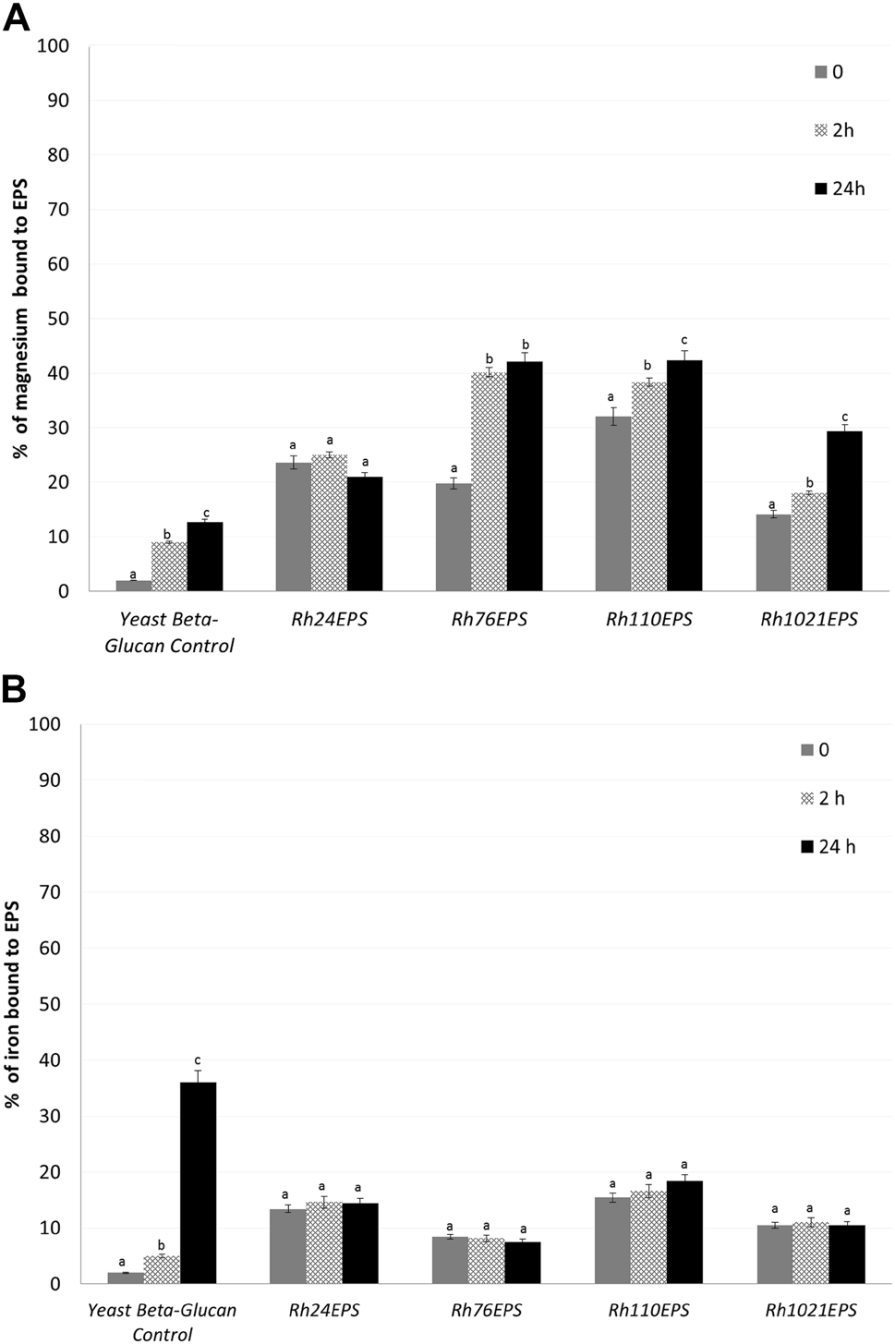
Assessment of the ability of exopolysaccharides from *R. leguminosarum* bv. *trifolii* Rt24.2 (*Rh24*EPS), *B. elkanii* USDA76 (*Rh76*EPS), *B. japonicum* USDA110 (*Rh110*EPS), and *S. meliloti* Rm1021(*Rh1021*EPS) strains to bind magnesium (**a**) and iron ions (**b**) during EPS incubation with human serum (2 and 24 h). The binding ability is expressed as a percentage of the tested substances bound to exopolysaccharides. All results are expressed as mean ± SD from three experiments (*n* = 3); values marked with different letters are significantly different (*p* ≤ 0.05)

The content of magnesium ions Mg(II) in the serum sample containing the tested EPS was assessed using the xylidyl blue monoreagent test, whereas the method with ferrozine as a reaction substrate was used for determination of iron ions (II). The results are shown in Fig. 3a, b as the percentage value of ion-sorption to exopolysaccharides isolated from the studied *Rhizobium* strains compared with the control serum without EPS. The highest ability of magnesium ion binding (approx. 42%) was observed for EPSs isolated from the *B. japonicum* USDA110 and from *B. elkanii* USDA76 strains. In turn, the analyses of iron ion sorption showed low values for all the tested preparations (from 7.5 to 14.4%).

### Effect of bacterial exopolysaccharides on laccase biochemical properties

**Fig. 4:**
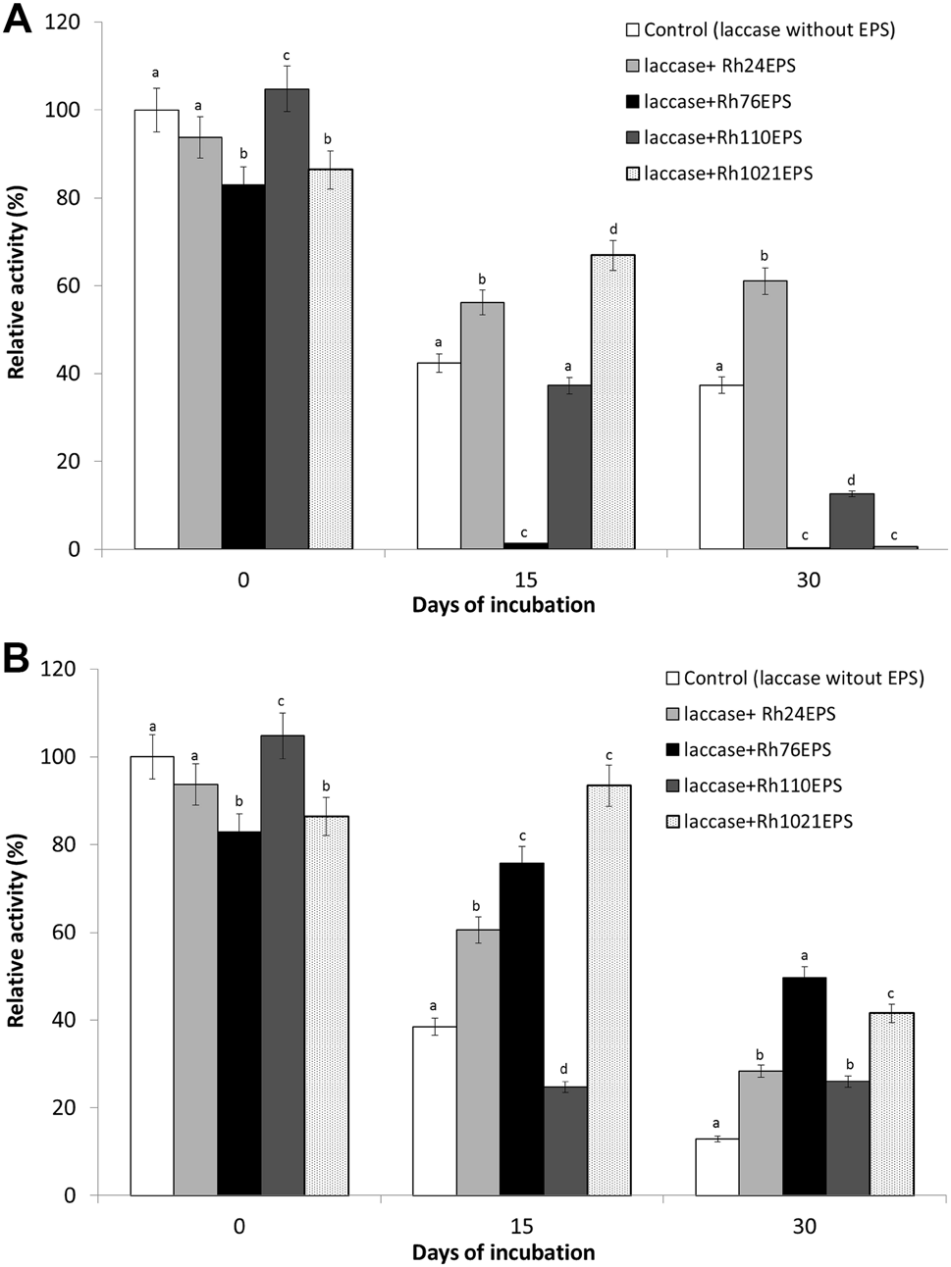
Effect of day incubation on laccase activity and stability in the presence of bacterial exopolysaccharides (*Rh24*EPS from *R. leguminosarum* bv. *trifolii* Rt24.2, *Rh76*EPS from *B. elkanii* USDA76, *Rh110*EPS from *B. japonicum* USDA110, and *Rh1021*EPS from *S. meliloti* Rm1021) at a temperature of 4 °C (**a**) and 25 °C (**b**). Experiments were performed in triplicate and relative activity was calculated from the activity at 4 °C and time of incubation 0. The average of the relative value (*n* = 3) and error bars are shown. Values with different letters are significantly different (*p* ≤ 0.05)

**Fig. 5:**
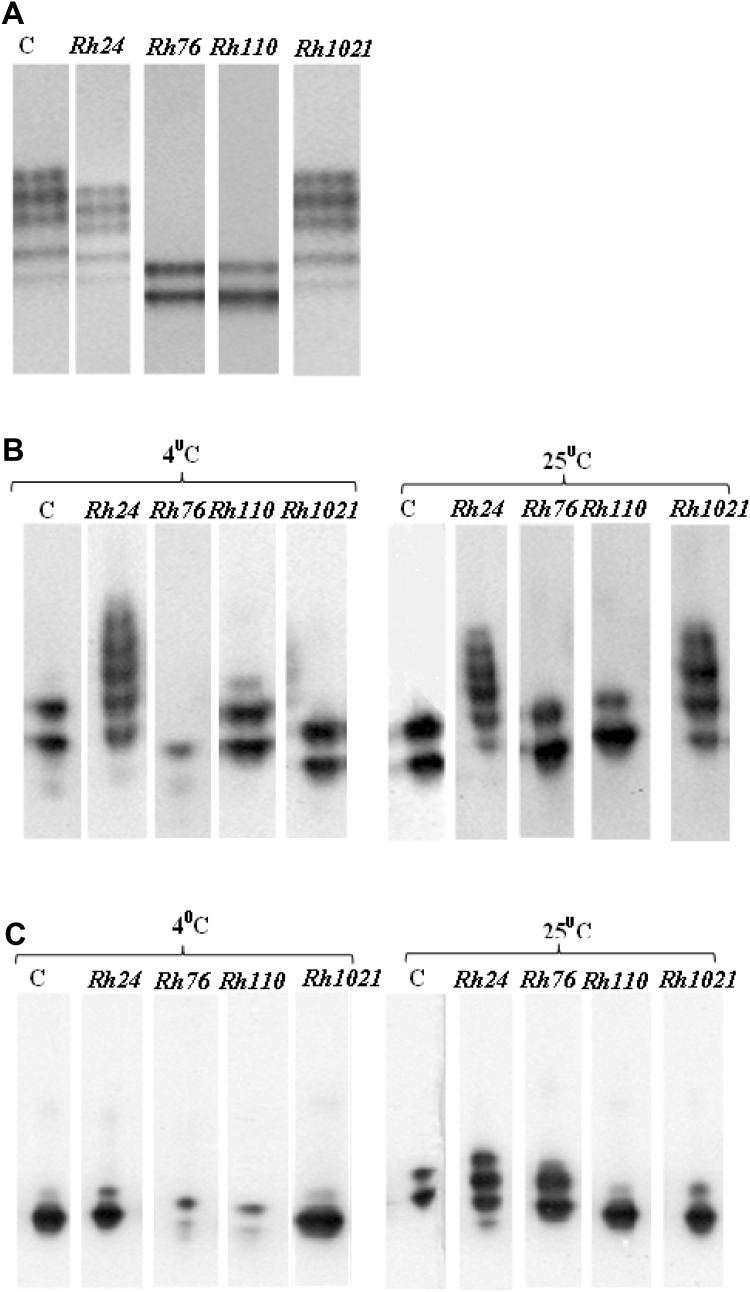
Native PAGE electrophoresis of laccase activities with bacterial exopolysaccharides: *Rh24*EPS from *R. leguminosarum* bv. *trifolii* Rt24.2, *Rh76*EPS from *B. elkanii* USDA76, *Rh110*EPS from *B. japonicum* USDA110, and *Rh1021*EPS from *S. meliloti* Rm1021. **a** Control (time of incubation 0), **b** electrophoresis of laccase activities after 15 days of incubation, and **c** electrophoresis of laccase activities after 30 days of incubation

The influence of bacterial exopolysaccharides on *C. unicolor* laccase activity and stability was determined. Laccase preparation was incubated in the presence or absence of *Rh*EPS for 15 and 30 days at temperatures of 4 and 25 °C. Incubation of the enzyme preparation and *Rh*EPS showed an influence of EPSs on the stability of the laccase compared to the control values (without EPSs). In the samples incubated without exopolysaccharides, significantly reduced laccase activity (only 37.3% of retained basal activity) was observed after 30-day storage of the enzyme at 4 °C. The results shown in Fig. 4a indicate that the presence of *Rh24*EPS in the probes preserves the laccase activity, since the retained enzyme activity was 61%. After incubation for 30 days at 25 °C, the laccase preparations incubated with all tested *Rh*EPS showed better catalytic activity compared to the control without EPSs (12.8% basal activity). The highest activity of laccases was obtained in the presence of *Rh76*EPS and *Rh1021*EPS, i.e., 49.5 and 41.5% of the initial catalytic activity, respectively (Fig. 4b). As shown in Fig. 5, the results presented above were also checked by native PAGE electrophoresis of laccase activities preincubated with bacterial polysaccharides. The conducted analysis has demonstrated that the effect on the profiles of laccase isoforms and intensification of activity bands depends on several variables in this experiment: the type of polysaccharide, incubation time, and incubation temperature. All these parameters induced very characteristic changes. At first, it was discovered that the electrophoretically detected laccase activity bands were considerably more intense in the samples stored at 25 °C in comparison to those kept at 4 °C after 15 and 30 days of incubations. Additionally, in the samples composed of laccase and *Rh24*EPS, the profile of the activity bands was practically not changed after 15 days of storage at both temperatures used; in the case of 25 °C, this result was similar also after 30 days of the experiment. A similar effect was also observed for the *Rh1021*EPS in the samples after 15 days of incubation at 25 °C. Contrary to this result, the initial profile of laccase activity bands was not preserved in the case of the control (without EPS addition) and the *Rh76*EPS or *Rh110*EPS containing preparations.

**Fig. 6:**
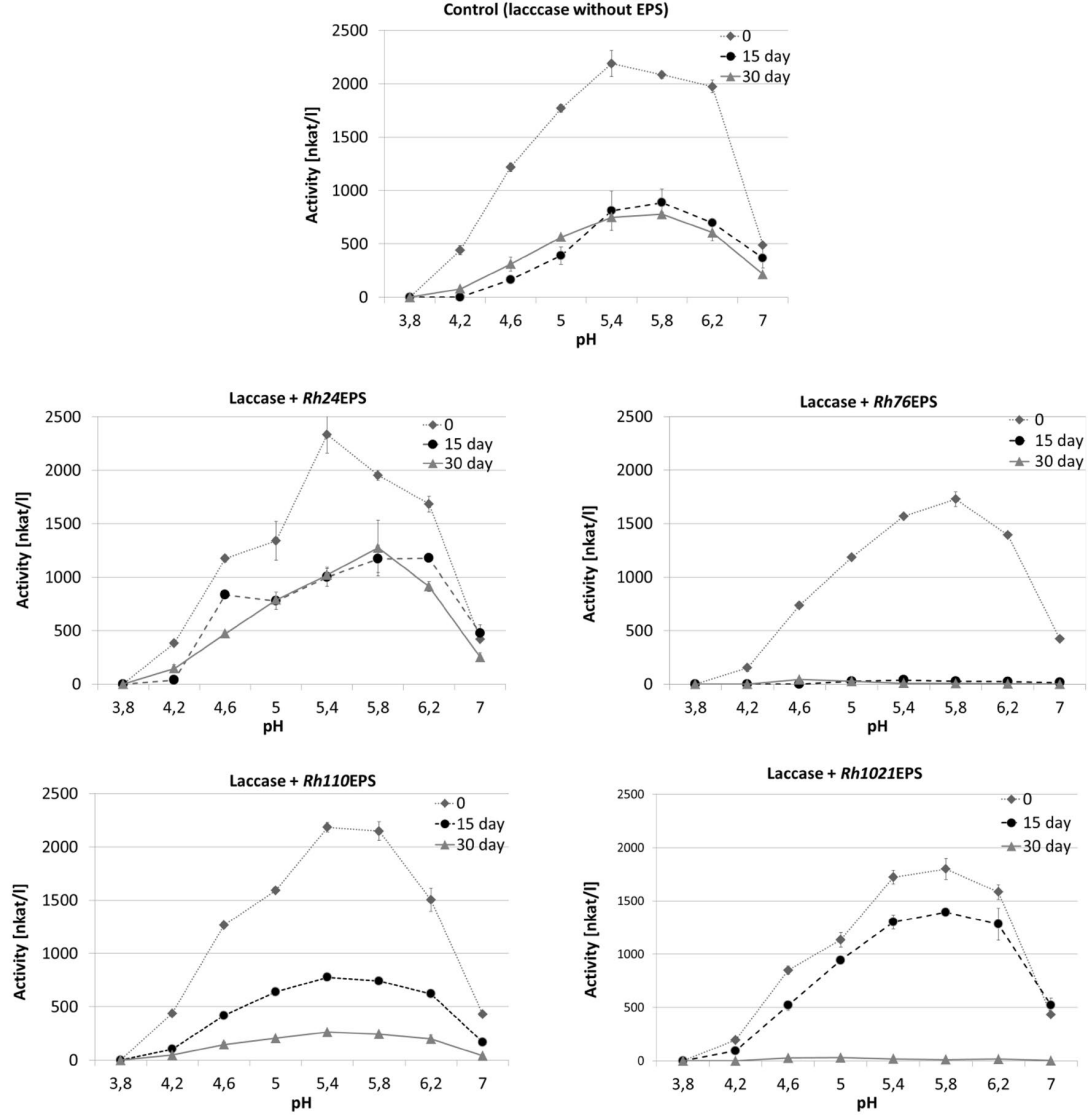
Effect of pH on laccase activity in the presence of bacterial exopolysaccharides: *Rh24*EPS from *R. leguminosarum* bv. *trifolii* Rt24.2, *Rh76*EPS from *B. elkanii* USDA76, *Rh110*EPS from *B. japonicum* USDA110, and *Rh1021*EPS from *S. meliloti* Rm1021 at a temperature of 4 °C, time of incubation 0, 15, and 30 days. Experiments were performed in triplicate and the average of relative value (*n* = 3) and error bars are shown. Values with different letters are significantly different (*p* ≤ 0.05)

**Fig. 7:**
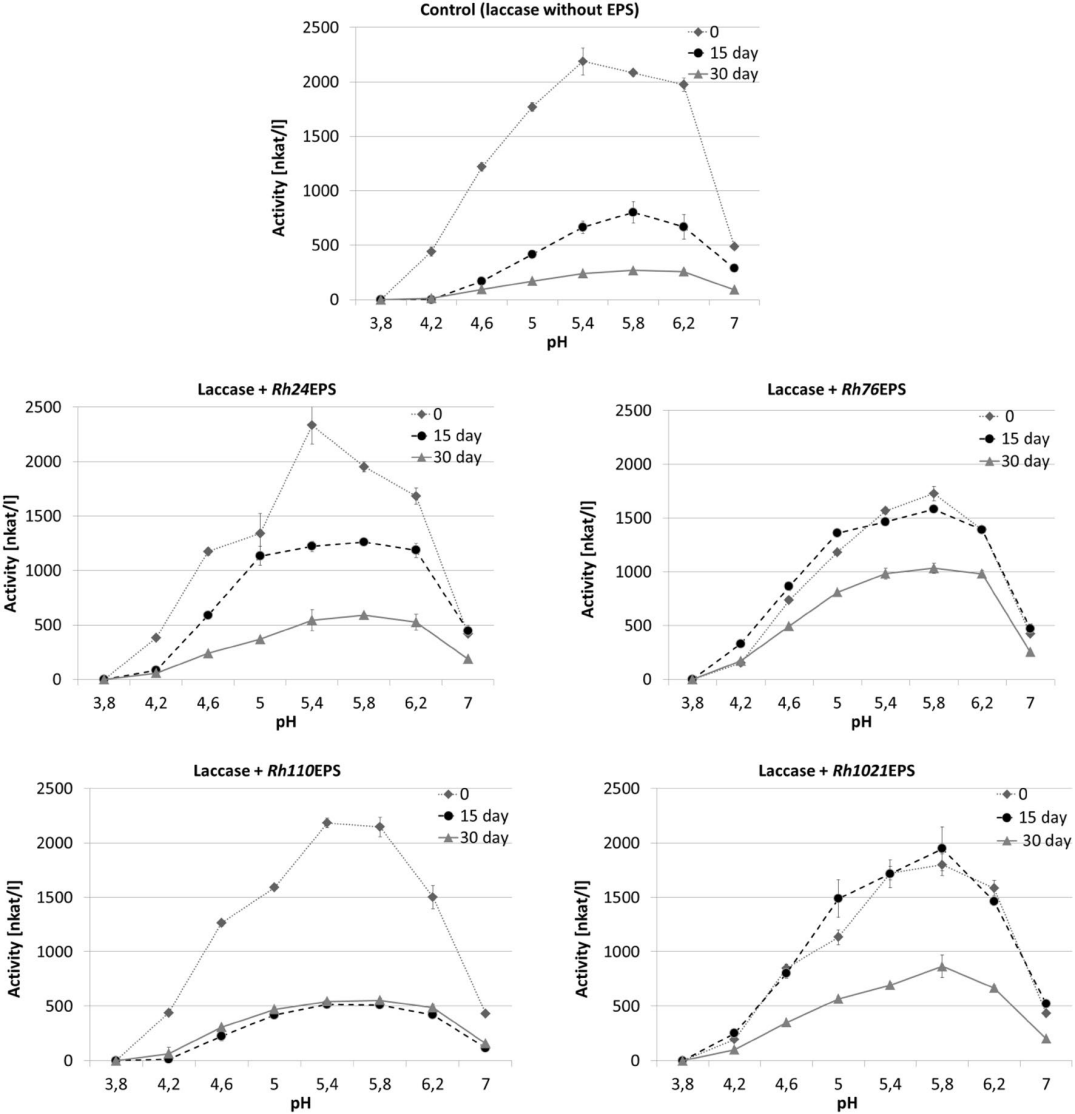
Effect of pH on laccase activity in the presence of bacterial exopolysaccharides: *Rh24*EPS from *R. leguminosarum* bv. *trifolii* Rt24.2, *Rh76*EPS from *B. elkanii* USDA76, *Rh110*EPS from *B. japonicum* USDA110, and *Rh1021*EPS from *S. meliloti* Rm1021 at a temperature of 25 °C, time of incubation 0, 15, and 30 days. Experiments were performed in triplicate and the average of relative value (*n* = 3) and error bars are shown. Values with different letters are significantly different (*p* ≤ 0.05)

The influence of the varying pH values (from 3.8 to 7.0) at 4 and 25 °C on laccase activity in the control samples without EPS and in the presence of bacterial polysaccharides was also estimated (Figs. 6, 7). Compared with the control, laccase activity in the preparation with *Rh1021*EPS showed no significant change after the first 15 days of incubation at 25 °C; however, after 15 consecutive days, the enzyme activity value decreased by approx. 50%. Similar results were obtained for laccase incubated in the presence of *Rh76*EPS but only at 25 °C (Fig. 7). In the presence of *Rh24*EPS, laccase activity showed a gradual decrease after 15 and 30 days of incubation at 25 °C (1200 and 505 nkat/l, respectively). In the presence of *Rh24*EPS and *Rh110*EPS, the control samples and enzyme preparations had similar pH activity profiles with the optimum activity at pH 5.4 (Figs. 6, 7). The most significant changes in the pH optimum (pH 5.8) were observed in the preparation of laccase with *Rh76*EPS and *Rh1021*EPS.

### Laccase kinetics

Kinetic parameters of the laccases (with syringaldazine as a reaction substrate) in the presence of EPSs were studied by determination of *K*_m_ and *k*_cat_ values (Table 2). Different redox activity of the laccases in the presence of bacterial EPSs has been shown. At time 0, laccase samples with *Rh76*EPS and *Rh110*EPS showed similar values to the control, while in the presence of *Rh24*EPS, the laccase *K*_m_ values (0.732 ± 0.05) was two times higher compared to the control. The changes in the *K*_m_ were not significant for all tested samples after 30 days of incubation at 25 °C, whereas *k*_cat_ was approximately two times lower for laccases in the presence of *Rh24*EPS (1.06 ± 0.03) and *Rh110*EPS (0.95 ± 0.02) compared to the control. In turn, there were no significant differences in the laccase *k*_cat_ values in the presence of *Rh76*EPS. For samples incubated at 4 °C the lowest *k*_cat_ value was obtained after 30 days of incubation for laccases with *Rh1021*EPS (0.062 ± 0.03) and *Rh76*EPS (0.165 ± 0.06). The highest laccase *k*_cat_ value was 1.129 ± 0.03 in the presence of *Rh24*EPS.

**Table 2:** Comparison of *K*_m_ and *k*_cat_ for laccase in the presence of bacterial EPS

	Day of incubation					
	0		15		30	
	*K*_m_ (mM)	*k*_cat_ (s^−1^)	*K*_m_ (mM)	*k*_cat_ (s^−1^)	*K*_m_ (mM)	*k*_cat_ (s^−1^)
(A) Temperature of incubation 4 °C						
Control (laccase without EPS)	0.338 ± 0.03	9.34 ± 0.1	0.73 ± 0.02	1.92 ± 0.02	0.266 ± 0.02	0.842 ± 0.02
*Rh24*EPS	0.732 ± 0.05	2.78 ± 0.09	0.704 ± 0.05	3.82 ± 0.1	0.267 ± 0.02	1.129 ± 0.03
*Rh76*EPS	0.337 ± 0.03	9.44 ± 0.2	0.732 ± 0.4	0.11 ± 0.05	0.286 ± 0.02	0.165 ± 0.06
*Rh110*EPS	0.308 ± 0.03	9.24 ± 0.2	0.731 ± 0.05	1.30 ± 0.03	0.267 ± 0.02	0.874 ± 0.06
*Rh1021*EPS	0.266 ± 0.02	7.87 ± 0.2	0.268 ± 0.02	3.24 ± 0.06	0.324 ± 0.03	0.062 ± 0.03
(B) Temperature of incubation 25 °C						
Control (laccase without EPS)	0.338 ± 0.03	9.34 ± 0.1	0.652 ± 0.03	2.445 ± 0.09	0.289 ± 0.03	2.36 ± 0.04
*Rh24*EPS	0.732 ± 0.05	2.78 ± 0.09	0.276 ± 0.02	1.952 ± 0.02	0.328 ± 0.03	1.06 ± 0.03
*Rh76*EPS	0.337 ± 0.03	9.44 ± 0.2	0.346 ± 0.03	2.205 ± 0.07	0.267 ± 0.02	2.79 ± 0.03
*Rh110*EPS	0.308 ± 0.03	9.24 ± 0.2	0.269 ± 0.02	0.335 ± 0.03	0.269 ± 0.02	0.95 ± 0.02
*Rh1021*EPS	0.266 ± 0.02	7.87 ± 0.2	0.268 ± 0.02	0.223 ± 0.02	0.332 ± 0.03	1.49 ± 0.01

All results are expressed as mean ± SD from three experiments (*n* = 3). The values within the columns followed by different letters are significantly different (*p* ≤ 0.05)

### Electrochemical parameters of laccase in the presence of bacterial EPS and EPS immobilized on carbon nanotubes

**Fig. 8:**
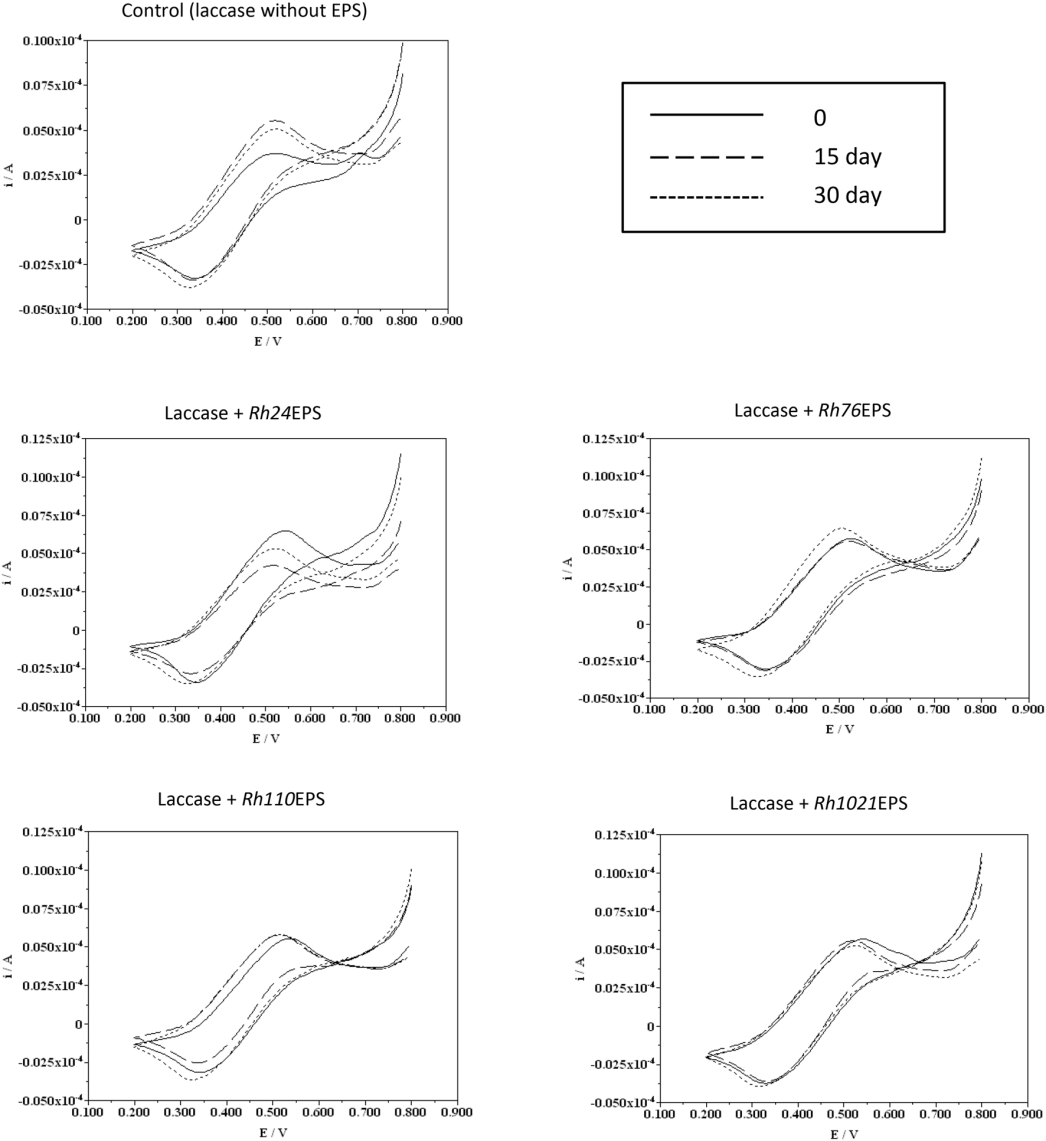
Electrochemical characterization of laccase in the presence of bacterial exopolysaccharides: *Rh24*EPS from *R. leguminosarum* bv. *trifolii* Rt24.2, *Rh76*EPS from *B. elkanii* USDA76, *Rh110*EPS from *B. japonicum* USDA110, and *Rh1021*EPS from *S. meliloti* Rm1021 incubated at a temperature of 4 °C, time of incubation 0, 15, and 30 days

**Fig. 9:**
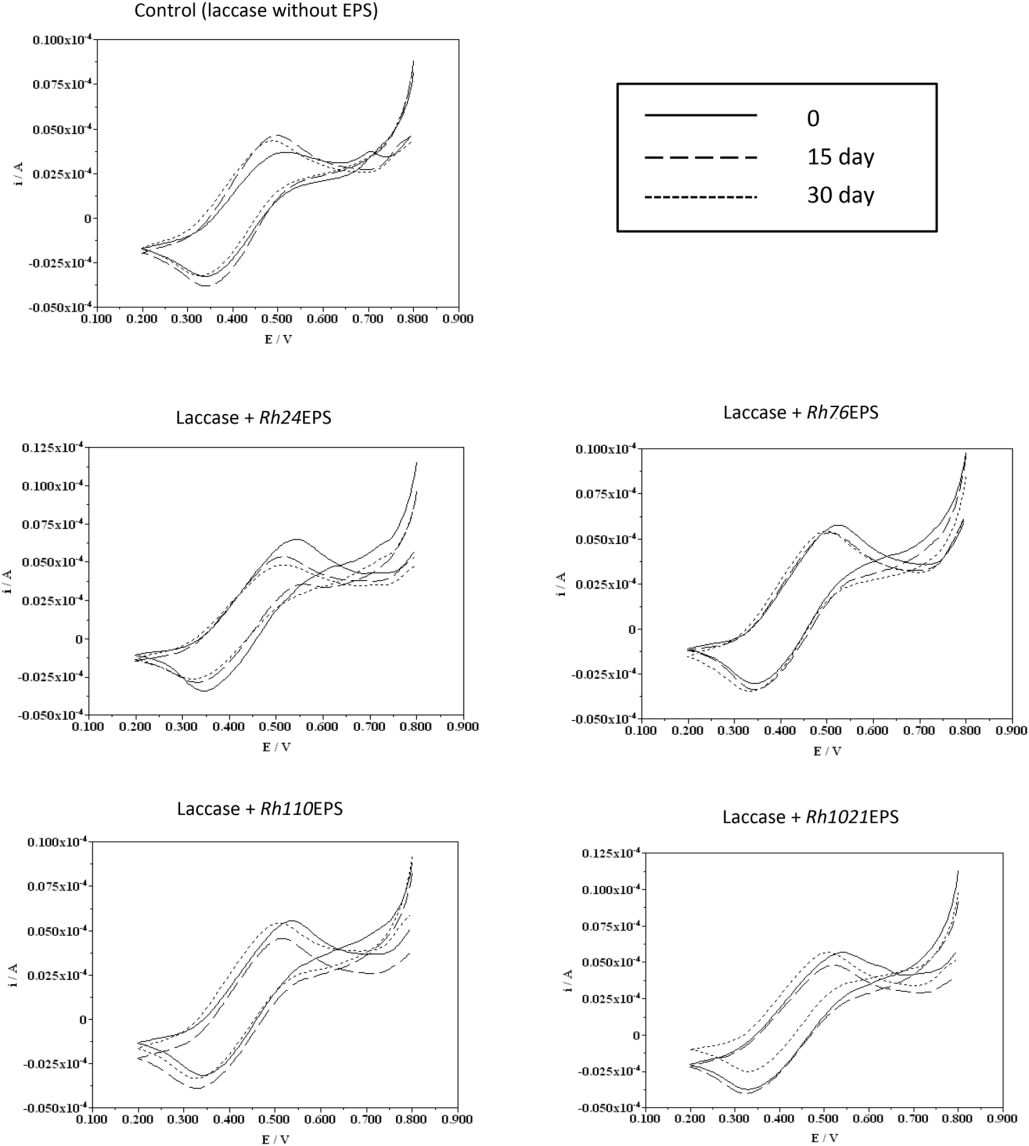
Electrochemical characterization of laccase in the presence of bacterial exopolysaccharides: *Rh24*EPS from *R. leguminosarum* bv. *trifolii* Rt24.2, *Rh76*EPS from *B. elkanii* USDA76, *Rh110*EPS from *B. japonicum* USDA110, and *Rh1021*EPS from *S. meliloti* Rm1021 incubated at a temperature of 25 °C, time of incubation 0, 15, and 30 days

Analysis of the electrochemical parameters of laccase was performed by cyclic voltammetry. Slow-scan voltammograms recorded in 0.2 mM substrate solutions (ABTS) pH 5 with laccase and bacterial EPS are shown in Figs. 8 and 9. The shape of these voltammograms is typical of catalytic redox processes taking place at the electrode–solution interface, which has been widely described in the literature. Cyclic voltammetry of the control laccase incubated at 4 °C showed slight differences between the anode and cathode potentials. Analysis of the laccase samples with EPS revealed that the presence of the polysaccharide in the reaction mixture results in a small potential shift in the direction of higher values. Major differences between the measurements of the tested samples and the control appeared in the current values. At time 0, laccase samples with *Rh110*EPS showed the most significant shift of the current values in the direction of higher values. Laccase samples with *Rh76*EPS, *Rh1021*EPS, and *Rh24*EPS had similar intensity values at all the test times. Cyclic voltammetry of the control sample after 15 and 30 days of incubation showed a shift towards higher current values. Voltammetric measurements of all tested laccase samples with rhizobial polysaccharides incubated at 25 °C showed little potential shifts toward higher values compared to the control, except the enzyme preparation with *Rh1021*EPS. The most significant displacement of the currents towards higher values (in the time 0) was obtained for laccases with *Rh110*EPS. Other tested samples showed similar intensity values at all the measurement times.

**Fig. 10:**
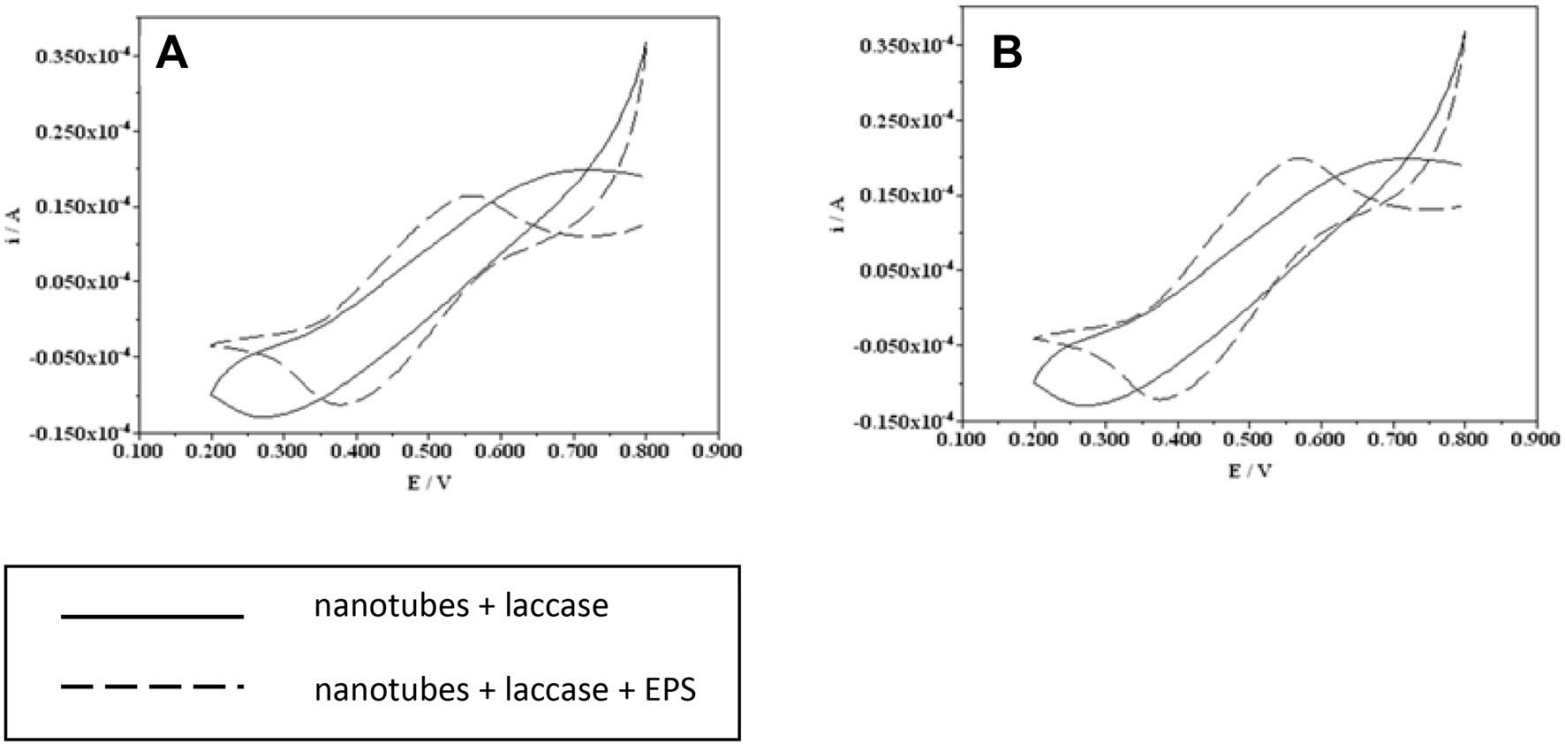
Electrochemical characterization of laccase (**a**) 10 µL carbon nanotubes + 10 µL laccases; 10 µL carbon nanotubes + 10 µl laccases with *Rh76*EPS, (**b**) 10 µL carbon nanotubes + 10 µL laccases; 10 µL carbon nanotubes + 10 µL laccases wit *Rh1021*EPS immobilized on the electrode working in the presence of a 0.2 mM solution of ABTS

Electrochemical analyses on an electrode with immobilized nanotubes were performed with the two samples of laccase with *Rh76*EPS and *Rh1021*EPS, which showed the greatest value of the application potential (Fig. 10). It was observed that the electrode with immobilized nanotubes exhibited pronounced peaks of oxidation and reduction obtained after the application of laccase with both EPSs. The laccases with bacterial polysaccharides had an evident shift of the potential value compared to the enzyme without addition of polysaccharides. The intensity values of the tested samples were similar to those recorded for the product without addition of laccase polysaccharides.

## Discussion

Exopolysaccharides are a diverse group of branched heteropolymer compounds consisting of multiple repetitive sugar subunits and secreted by microorganisms to the soil under extreme and stressful condition [1]. The chemical structure of the studied exopolysaccharides produced by bacteria belonging to four different species of rhizobia was described earlier [28–31]. In our study, analysis of the FT-IR spectrum of the ethanol-extracted exopolysaccharides of all bacterial strains confirmed their typical carbohydrate pattern. As shown in our previous studies, the investigated rhizobial EPSs obtained with the precipitation method with 96% ethanol are not toxic, have no antibacterial properties, and exert a low antioxidant effect. In addition, they are derived from non-pathogenic bacteria and, therefore, they are safe for humans [2, 28].

In addition, the microscopic visualization of the exopolysaccharides after staining with Fluorescence Brightener 28 showed the presence of β-linked bonds and showed their varied morphological structures. These results confirmed the varied chemical structure of all investigated compounds.

The ability to bind heavy metals by exopolysaccharides and a mixture of polysaccharides were described in the literature data. Cations can be bound by positively charged polysaccharides or in a reaction with hydroxyl groups [46]. Studies carried out on *Rhizobium etli* bacteria showed that capsular polysaccharide rapidly bound Mn (II) ions (62 mg Mn per g of CPS) and less readily Pb(II) and Cu(II) ions [47, 48]. In the present study, it was shown that EPS isolated from the *B. japonicum* USDA110, and *B. elkanii* USDA76 strains bound Mg(II) ions with a promising yield. This binding ability may probably be used in the modification of biocatalysis, the bioremediation process or in food industry but this subject requires further investigations.

Laccase is an enzyme with a very high application potential. Our recent reports suggest that laccase isolated from *C. unicolor* possesses also antiproliferative and antiviral properties [20]. It is known that one of the difficulties in the use of the enzyme in the industry is to keep its storage operative stability [49]. Therefore, numerous studies on laccase stability, both by the chemical modification of the enzyme molecule and by modifying the reaction conditions and the environment are conducted [23, 50, 51].

To stabilize soluble proteins, different excipients such as sugar, polymers, surfactants, and others are used. In the present study, four bacterial EPSs (*Rh24*EPS, *Rh1021*EPS, *Rh110*EPS and *Rh76*EPS) were investigated towards the modification of the operative stability of *C. unicolor* laccase and its biochemical properties. Polysaccharides have numerous bioactive properties, and are also believed that some of them can act as an effective stabilizer in the reaction medium [20, 52, 53]. For example, laccase linked with chitosan showed higher stability in a wider range of temperatures and pH compared to non-immobilized enzyme [54, 55]. Another compound often used in biosensors in combination with laccase is mucin [27]. Besides polysaccharides, organic solvent and trifluoromethanesulfonate ionic liquids have been used [49, 50].

The exopolysaccharides analyzed in the present paper have different chemical structures composed of repeated subunits and similar physicochemical properties that seem to be useful for laccase activity stabilization. Furthermore, these exopolysaccharides are derived from non-pathogenic bacteria, and therefore, they seem to be safe for humans. This is an important aspect especially in the context of the possibilities of their application in medicine. Since *S. meliloti* Rm1021, *B. japonicum* USDA110, *B. elkanii* USDA76, and *R. leguminosarum bv. trifolii* Rt24.2 bacteria [56] are efficient producers of EPS, the production process is cheap and fast. The effect of polysaccharides isolated from the bacterium *R. leguminosarum* bv. *trifolii* Rt24.2 on the stability and activity of lipase isolated from *Rhizomucor variabilis* was analyzed with satisfactory results by Bancerz et al. [10, 50]. In the present study, a positive effect of the presence of proposed polysaccharides in the reaction mixture on the stability of the laccase during 30-day incubation (especially at 25 °C) was detected. It seems that the *Rh76*EPS and *Rh1021*EPS preparations demonstrate the greatest application potential. The best stability of laccase activity was observed in this case (respectively, 49.5 and 41.5% of relative activity) compared to the control without polysaccharides (12.8%). Incubation of laccases with *Rh76*EPS and *Rh1021*EPS contributes to significant changes in the optimum pH of the enzymatic reaction towards more alkaline values. Similar results were obtained by Bancerz et al. [10] in their study of lipases in the presence of bacterial (from *R. leguminosarum* bv. *trifolii* Rt24.2) and fungal (from *Ganoderma applanatum*) polysaccharides. In the case of laccase properties, an important aspect may be the interaction between the polysaccharides used and the stabilization and binding of copper ions, which are the catalytic element of the active center of this enzyme. It may directly modulate its properties, especially operation stability. Considering the absorption potential of the described components in relation to divalent metal ions such as magnesium, this type of action seems to be possible.

The effect of *Rh24*EPS and *Rh1021*EPS on the preservation of the laccase isozyme profile may probably be connected with some proteolytic remodeling of laccase subunits described by Janusz et al. [57]. One of the hypotheses is that the probable crosslinking interactions between the laccase and EPS molecules inhibit the maturation of the enzyme. Another possibility can also be the modification of the protein surface by the tested EPS. This can influence its overall charge and different migration profile especially under native PAGE condition.

Increased substrate specificity of the enzyme and its higher yield of catalytic properties are desired in biocatalysts used in the industry and biotechnology. In our study, an increased *kcat*/*Km* value, used to assess the catalytic laccase efficiency [58], was observed in the samples with *Rh76*EPS and *Rh1021*EPS. Therefore, it can be assumed that the bacterial EPSs used in the present work can not only stabilize laccase under extreme reaction conditions (pH and temperature range, and long-term storage of an aqueous solution of the enzyme) but also significantly modified the biocatalytic properties of this enzyme.

The results of the experiments indicate that the incubation of laccase with the selected polysaccharides has shown significant effects on the electrochemical parameters of the tested biocatalyst. In our study, an increase in the current in the nanotubes modified by the laccases with the rhizobial EPSs used was observed, compared to the control without polysaccharides. This process indicates a positive influence of the connection between polysaccharides and *C. unicolor* laccase on the oxidation and reduction reactions in the described electrode system. An increase in the efficiency of the electrochemical measurement system with the carbon nanotubes was reported by Stolarczyk et al. [59]. The improved electrochemical measurement system allows a direct electron exchange between the electrode surface and the enzyme active center and is, therefore, widely used in the construction of biosensors [59].

## Conclusion

In this study, rhizobial exopolysaccharides were used for the first time for the modification of *C. unicolor* laccase properties, especially storage stability. Given the improvement of the biochemical properties of the laccases in the presence of the EPSs applied, especially at elevated temperatures and a broad range of pH, this experimental system can be proposed as a promising tool in biotechnological applications in textile, paper, food, cosmetic industries, in the bioremediation process as well as medicine and environmental protection. The bacterial EPSs used as natural stabilizers increasing the electrochemical properties of laccases can be very promising in, e.g., biosensor constructions. In addition, the metal ion binding ability of the described bacterial polysaccharides in the context of enzyme activity seems to be very promising in practical uses but requires further detailed research.
